# Whole-genome sequencing from the New Zealand *Saccharomyces cerevisiae* population reveals the genomic impacts of novel microbial range expansion

**DOI:** 10.1093/g3journal/jkaa027

**Published:** 2020-12-22

**Authors:** Peter Higgins, Cooper A Grace, Soon A Lee, Matthew R Goddard

**Affiliations:** 1 The School of Life Sciences, College of Science, University of Lincoln, Brayford Pool, Lincoln, LN6 7TS, UK; 2 Department of Biology, York Biomedical Research Institute, University of York, Heslington, York, YO10 5DD, UK; 3 Department of Biological and Geographical Sciences, University of Huddersfield, Queensgate, Huddersfield, HD1 3DH, UK; 4 The School of Biological Sciences, University of Auckland, Auckland 1142, New Zealand

**Keywords:** Saccharomyces cerevisiae, New Zealand, Population Genomics, Range Expansion, Microbial Ecology

## Abstract

*Saccharomyces cerevisiae* is extensively utilized for commercial fermentation, and is also an important biological model; however, its ecology has only recently begun to be understood. Through the use of whole-genome sequencing, the species has been characterized into a number of distinct subpopulations, defined by geographical ranges and industrial uses. Here, the whole-genome sequences of 104 New Zealand (NZ) *S. cerevisiae* strains, including 52 novel genomes, are analyzed alongside 450 published sequences derived from various global locations. The impact of *S. cerevisiae* novel range expansion into NZ was investigated and these analyses reveal the positioning of NZ strains as a subgroup to the predominantly European/wine clade. A number of genomic differences with the European group correlate with range expansion into NZ, including 18 highly enriched single-nucleotide polymorphism (SNPs) and novel *Ty1/2* insertions. While it is not possible to categorically determine if any genetic differences are due to stochastic process or the operations of natural selection, we suggest that the observation of NZ-specific copy number increases of four sugar transporter genes in the *HXT* family may reasonably represent an adaptation in the NZ *S. cerevisiae* subpopulation, and this correlates with the observations of copy number changes during adaptation in small-scale experimental evolution studies.

## Introduction

Most plant and animal species have defined geographic ranges that they inhabit. The invasion of ecologically similar but geographically different habitats, to which species are reasonably well preadapted, may occur if migration and the absence of competition allows. The invasion of new ranges by species can have major impacts on genetic diversity and population structure (Braga *et al.* 2019), including admixture, adaptive evolution, and neutral evolution via genetic drift and bottleneck events (Keller *et al.* 2010; Swaegers *et al.* 2015; *e.g.*Garcia-Elfring *et al.* 2017). The population genetic effects of range expansion have been reported in both plant species, *e.g.* accumulation of deleterious mutations in *Mercurialis annua* (González-Martínez *et al.* 2017), and animal species, *e.g.* poleward expansion in the damselfly *Coenagrion scitulum* (Swaegers *et al.* 2015). However, the concept of range expansion in microbes has only been more recently explored, and its impact on microbial population structure and evolution is not as well understood. Most studies have focused on pathogenic species – primarily plant-pathogenic fungi (for review, see Thakur *et al.* 2019). Like many other aspects of microbial ecology, range expansion of microbes is poorly characterized due to their cryptic nature (Jarić *et al.* 2019). It is now clear that Baas-Becking’s classic microbial biogeographic hypothesis of “*Everything is everywhere:* but *the environment selects*” does not hold universally for microbes (van der Gast 2015). While some microbes appear to be dispersed ubiquitously, others have restricted distributions, as described by a moderate endemicity model of microbial biogeography (Foissner 2007). This suggests that, as with macroscopic species, geographic separation has the potential to drive population structure alongside environmental processes (O’Malley 2008). However, the factors that constrain or define microbial range are not always clear, and the relative contribution of ecological and geographical factors is still being evaluated (Power *et al.* 2018; Meyer *et al.* 2018).

*Saccharomyces* are unicellular diploid eukaryote fungi that undergo sexual reproduction, and these yeasts have a long historical association with human populations due to their ability to produce CO_2_ and ethanol via sugar fermentation. *Saccharomyces cerevisiae* has been involved in the production of fermented beverages for at least 9000 years (McGovern *et al.* 2004). The global phylogeny of *S. cerevisiae*, based on partial and whole-genome sequencing, shows it to be grouped both by technological origins (including wine, beer, bread, cheese, rum, and sake) and geographic area (European, North American Oak, West Africa, Malaysia, and the Far East) (Legras *et al.* 2007; Liti *et al.* 2009; Wang *et al.* 2012; Peter *et al.* 2018). The general picture is that *S. cerevisiae* originated in Far East Asia (Wang *et al.* 2012; Duan *et al.* 2018) and has expanded and diversified to now comprise specific geographic subpopulations. However, it is not clear what forces maintained these geographic subpopulations. In addition, several distinct lineages have been inadvertently domesticated by humans for food and beverage fermentations from these subpopulations (Duan *et al.* 2018; Steensels *et al.* 2019). Among the most studied of these are the *S. cerevisiae* strains associated with viticulture and winemaking. Recent work has shown this group to be domesticated from a wild population inhabiting the Mediterranean region, presumably alongside the development and expansion of viticulture there (Almeida *et al.* 2015). As viticulture has since spread throughout the world, this group of vine and wine-associated yeasts (the wine group) were brought with it.

Population structure and genetic diversity of the *S. cerevisiae* wine group has been associated with geographic location at global and regional scales (Martínez *et al.* 2007; Viel *et al.* 2017), grape variety (Schuller *et al.* 2012), and environmental conditions, *e.g.* freeze-thaw resistance and copper sulfate resistance (Kvitek *et al.* 2008). Research has indicated the diversity of the wine group originates from dispersal and expansion into new environments due to the novel selection pressures encountered, such as metabolic stress in industrial environments (Dunn *et al.* 2012). Adaptive variation may include gene gain or loss (Almeida *et al.* 2017), mutations within genes (Aa *et al.* 2006), and copy number variation of genes to mediate regulation of gene products (Dunn *et al.* 2012). However, it has also been suggested that the genetic differences observed between *S. cerevisiae* populations may be due to neutral processes, primarily population bottlenecks and genetic drift (Warringer *et al.* 2011). This view has been supported by a number of studies (Liti *et al.* 2009; Zörgö *et al.* 2012) and forms the basis of the neutral nomad model of *S. cerevisiae* (Goddard and Greig 2015). Here, the genetic diversity of *S. cerevisiae* is explained as neutral divergence through isolation by distance, as observed by the lack of gene flow between geographically remote populations. It seems highly probable that both neutral and adaptive processes have contributed to the genetic architecture of modern *S. cerevisiae* lineages, but establishing their relative contributions is difficult without further understanding the life history of these lineages. Before making ecological inferences about genomic features, it is vital to ascertain whether specific genomic features have arisen through adaptive or neutral processes.

As one of the last landmasses colonized by humans, ∼700 years ago (King 2003), and one of the most geographically isolated, New Zealand (NZ) provides a unique opportunity to study the effects of range expansion for species generally and is thus also ideal to evaluate microbial range expansion. Extensive sampling in NZ vineyards, wineries, and native forests has discovered approximately 2000 genotypes of *S. cerevisiae* comprising regionally distinct subpopulations at scales greater than 1000 km, inferred using variance at microsatellite loci (Goddard *et al.* 2010; Knight *et al.* 2015). Microsatellite and Rad-Tag DNA sequence (Cromie *et al.* 2013) analyses suggest that the NZ population is closely related to, but distinct from, the wine group, whereas analyses of 106 conserved loci derived from 50 whole-genome sequences of these isolates infers these to be mostly interdigitated with the wine group, and this group expanded to NZ via human-aided dispersal within only the last 1000 years (Gayevskiy *et al.* 2016). The status of the NZ population is thus not clear.

Transposable elements (TEs) are mobile components of almost all eukaryotic genomes, and their evolutionary history typically follows that of their hosts (Bowen *et al.* 2003; Hosid *et al.* 2012). *S. cerevisiae* contains multiple families of the TE subclass known as long-terminal repeat (LTR) retrotransposons, named *Ty1-5* (Kim *et al.* 1998), that drive their own replication and integration into the genome via an RNA intermediate. LTR sequences frame *gag* and *pol* open reading frames (ORFs), which encode proteins necessary for their transposition (reviewed by Havecker *et al.* 2004). LTR sequences are identical upon transposition, and as such present a target for homologous recombination, which results in the deletion of the ORFs and one LTR, leaving a “solo” LTR remnant in the genome (Lesage and Todeschini 2005). Hybridization between the closely related elements of the *Ty1-2* superfamily is also thought to occur via recombination (Jordan and McDonald 1999; Czaja *et al.* 2020). Analysis of the complex familial histories by Carr *et al.* (2012) revealed that elements of the *Ty1-2* superfamily are still highly active, members of *Ty3* and *Ty4* transpose at a relatively low rate and *Ty5* is extinct in *S. cerevisiae*. Insertion sites of these elements have therefore been used to infer evolutionary relationships in *S. cerevisiae*, with insertions shared between populations providing strong evidence for common ancestry and horizontal transfer from other species providing evidence of geographical background and life histories (Carr *et al.* 2012). Bleykasten-Grosshans *et al.* (2013) analyzed the *Ty* insertion profiles of geographically and ecologically diverse strains of *S. cerevisiae to* identify the patterns of *Ty* distribution, which contribute to their diversity. However, little investigation into the evolutionary history of *Ty* elements in an isolated population such as NZ has been conducted.

In this study, an additional 52 *S. cerevisiae* genomes are sequenced from the NZ population, and combined with the existing NZ derived data to total 104 high-quality genomes. Here, the entirety of genomes (as opposed to select loci used in previous studies (Gayevskiy *et al.* 2016)) is used to evaluate the micro-evolutionary nature of microbial range expansion into NZ. Signals of differentiation are examined to determine the effects of range expansion on *S. cerevisiae* genetic diversity and population structure. The origins of these signals are investigated, and we attempt to infer if these are from natural selection, or if divergence within NZ is primarily due to neutral processes. These analyses will also allow the placement of NZ *S. cerevisiae* within the wine clade to be more accurately estimated.

## Methods

### Strain selection

The sampling locations of the 104 strains were across major wine growing areas on NZ’s north and south islands (Supplementary Table S1), and all but one strain derived from spontaneous wine ferments or vineyard habitats; the one exception was isolated from native forest soil. Fifty-two unsequenced strains were selected to complement those genomes analyzed by Gayevskiy *et al.* (2016) (originally reported in Goddard *et al.* 2010; Knight and Goddard 2015). Each strain was propagated in YPD, and high molecular weight genomic DNA was extracted using the Qiagen Blood & Cell Culture DNA Kit. Libraries were constructed using the Illumina TruSeq Nano DNA Sample Prep Kit with 550 bp insert size. Sequencing was carried out at the Beijing Genomics Institute (China) on three 150-bp paired-end lanes of an Illumina HiSeq 2000.

### Genome sequencing and read alignment

FASTQC v0.11.7 (Andrews 2010) was used to analyze the quality of libraries and identify trimming parameters. Reads were trimmed using Trimmomatic v0.36 (Bolger *et al.* 2014) with parameters LEADING:3 TRAILING:3 SLIDINGWINDOW:3:20 MINLEN:30 and ILLUMINACLIP to remove adapter sequences. Resulting trimmed reads were compared to raw reads to confirm quality improvement. Trimmed reads were aligned to the reference strain S288C using Bowtie2 v2.3.4.1 (Langmead and Salzberg 2012). Aligned Bam files were sorted and aligned using Samtools v1.8 (Li *et al.* 2009). Duplicate reads were removed with Samtools markdup function. Variants were called with bcftools mpileup with default parameters, and bcftools call with parameters -mv –ploidy 2. The Samtools package bcftools filter was used to remove reads with quality scores less than 20.

### Population structure analysis

Prior to structural analysis, samples were filtered to reduce computational time and remove uninformative SNPs. Using vcftools v0.1.16 (Danecek *et al.* 2011) with flags –maf 0.02 –max-maf 0.98 –max-missing-count 0 –thin 50, rare SNPs, SNP sites missing data in any sample and any SNPs within 50 bp of each other were removed. PLINK v1.90b4 (Purcell *et al.* 2007) variant pruning was used with –indep-pairwise 50 5 0.9 and –hwe 1e−10 to filter out SNPs with high linkage disequilibrium (LD) and those far from Hardy–Weinberg equilibrium. To identify population structure within NZ, Structure v2.3.4 (Pritchard *et al.* 2000) was run using both Admixture and Linkage models on all 104 strains. Values for the number of assumed clusters (*K*) were tested from 2 to 12, with five repeat runs at each value of *K*. Population classifications were not used for the prior. Iteration numbers were increased until convergence was achieved for all values of *K*. The resulting Structure data were analyzed in CLUMPAK v1.1.2 (Kopelman *et al.* 2015) to test for group modality and aligning results over all values of *K*, and the Evanno method was employed to determine the optimal value of *K* (Evanno *et al.* 2005). To test for biogeographical structure, Structure output for the optimal *K* value was analyzed in ObStruct v1.0 using either sampling locations or North/South Island as predefined populations (Gayevskiy *et al.* 2014). An additional Structure analysis was conducted using the Admixture model on 15272 SNPs from a randomized selection of 50 NZ strains, 40 EU strains—including 5 from each defined subpopulation—and 5 from both alpechin and mosaic populations, for *K* values of 1–4.

### Analysis of variant SNPs

SNP data for published strains were taken from Peter *et al.* (2018) with SNPs extracted from the provided gvcf file. Data for 362 wine-group strains were extracted and combined with NZ vcf files before conversion to gds format using gdsfmt v1.22.0 (Zheng *et al.* 2012). The combined dataset was filtered to remove SNPs with missing rates greater than 0.9 and with LD greater than 0.5. In addition, the NZ data were filtered similarly for use in independent analysis. Principal component analysis (PCA) was performed on both the NZ data and the combined dataset using snpgdsPCA in SNPRelate v1.20.1 (Zheng *et al.* 2012). Samples were assigned to populations based on Structure results for *K* = 2, 3 and 4. A sample was assigned to a population if it had greater than 0.8 inferred ancestry from that population. Samples with less than 0.8 inferred ancestry from any population were designated as admixed. Using the SNP loadings from this PCA, the wine-group strains were projected onto the NZ Principal Components with functions snpgdsPCASNPLoading and snpgdsPCASampLoading. In addition, PCA was performed on the combined wine-NZ dataset. Two phylogenies were generated: the first included all strains from Peter *et al.* (2018) and all 104 NZ samples and the second included only NZ, wine-group, and closely related mosaic and alpechin (olive mill wastewater) strains. Phylogenies were created using hierarchical clustering in SNPRelate and visualized with iTOL v4 (Letunic and Bork 2019).

### Signals of SNP selection

To model differences in SNP frequency, BayeScan v2.0 was used on a subset of SNPs from both NZ and wine-group strains, filtered with vcftools to remove rare SNPs (those with minor or major allele frequencies below 10%) (Foll and Gaggiotti 2008). BayeScan estimates the relative posterior probabilities for each locus being under either diversifying selection or balancing/purifying selection. Multiple runs of BayeScan were conducted with varying model parameters and the results of each run were analyzed to test for convergence, as confirmed with Gelman and Rubin’s diagnostic in R package coda v0.19.3 (Plummer *et al.* 2006).

### TE analysis

One hundred four NZ genomes were screened with RepeatMasker (Smit *et al.* 2013) for the presence of *Ty1-5* LTRs using a custom library (see Supplementary File S5). The genomic co-ordinates generated by RepeatMasker were converted to .bed format and used to extract full-length LTR sequences from contigs of each strain using Bedtools getfasta (Quinlan and Hall 2010). Co-ordinates were modified in R (R Core Team 2020) to include 5-bp up/downstream to obtain target site duplications (TSDs) present at the 5′ and 3′ sequence boundaries. Partial LTRs and those whose TSDs could not be determined, *i.e.* due to the presence of Ns or placement at the end of a contig, were discounted from analysis. NZ LTRs were sorted by superfamily and added to modified superfamilial LTR datasets compiled by Carr *et al.* (2012). Carr *et al.*’s (2012) datasets were obtained by screening the *Saccharomyces* Genome Resequencing Project (SGRP) *S. cerevisae* and *Saccharomyces paradoxus* genomes (available at https://www.sanger.ac.uk/research/projects/genomeinformatics/sgrp.html, last accessed 2nd January 2021). The publicly available genomes of *Saccharomyces kudriavzevii* (assembly AACI03), *Saccharomyces eubayanus* (assembly TUD_Seub_ont_v2), *Saccharomyces uvarum* (assembly AACA01; formerly *Saccharomyces bayanus*), and *Saccharomyces mikatae* (assembly AACH01) were also screened for LTR sequences. LTRs were aligned with MAFFT v7.310 (Katoh and Standley 2013) using the FFT-NS-2 alignment method and –leavegappyregion as an additional parameter. Alignments were trimmed with trimAL v.1.2 (Capella-Gutiérrez *et al.* 2009) with the options -gt 0.9 and -cons 90 (with the exception of the *Ty1-2* alignment, which used the option -cons 70). Identical sequences and LTRs shared between multiple strains (as determined by TSDs) were removed, with preference given to the SGRP strains. Alignments for each superfamily were converted to PHYLIP format for phylogenetic analysis with RaxML-HPC2 (Stamatakis 2014) on XSEDE as part of the CIPRES Science Gateway (Miller *et al.* 2010). The following parameters were used: GTRGAMMA nucleotide bootstrapping model; 1000 bootstrap iterations; random seed values for parsimony and rapid bootstrapping. All other parameters were kept as default. Resulting trees were visualized with FigTree v. 1.4.4 (Rambaut 2012).

### Analysis of Copy Number Variants

To search for signatures of selection, NZ genomes were mapped to the published *S. cerevisiae* ORF pangenome using bwa v0.7.17 with the -U 0 flag set, so as to not penalize unpaired reads due to the shorter targets (Li and Durbin 2009). This ORF pangenome was constructed using de novo assembly of 1011 *S. cerevisiae* genomes and consists of 7796 ORFs, with closely related and duplicate sequences removed (Peter *et al.* 2018). The copy number of each ORF was determined by dividing the median read depth at each ORF by the median read depth for that sample. These results were compared to the ORF copy numbers given for 362 strains in the wine group. Median copy numbers were calculated for each population. ORFs were filtered to include only those with a median copy number of at least 0.5 in either population. Those ORFs enriched in NZ by a factor of 2 or greater were selected for Gene Ontology enrichment analysis. GOrilla was used to search for enriched Process, Function and Component gene ontology (GO) terms against a background of all verified *S. cerevisiae* genes (Eden *et al.* 2009). The presence/absence of the 2 μ plasmid and its relative copy number were calculated as the median of plasmid ORF copy numbers.

### Data availability

Raw sequence data have been made publicly available at the NCBI Sequence Read Archive with accession PRJNA649809. Supplementary material is available at figshare DOI: https://doi.org/10.25387/g3.12820517. Supplementary Figure S1 shows hierarchical clustering analysis for 1115 *S. cerevisiae* strains. Supplementary Figure S2 shows Evanno method results for determining *K*. Supplementary Figures S3 and S4 show phylogenies for Ty3 and Ty4. Supplementary Files S1–S4 are tree files for the Ty family. File S5 is a custom Ty library. Supplementary Table S1 shows sampling information. Supplementary Table S2 includes a list of enriched ORFs in NZ. Supplementary Table S3 shows additional Gene Ontology details.

## Results

In total, 346 million 150-bp paired-end reads were obtained, giving an average of 6.7 million reads per genome. Of these, an average of 95.3% mapped to the S288C reference genome, with an average read depth of 63.9X over all covered regions and are thus comparable to those genomes generated by Gayevskiy *et al.* (2016). When combined with the data from Gayevskiy *et al.* (2016), 604 million mapped reads underlie the 104 genomes.

### Global positioning of NZ isolates

**Figure 1:**
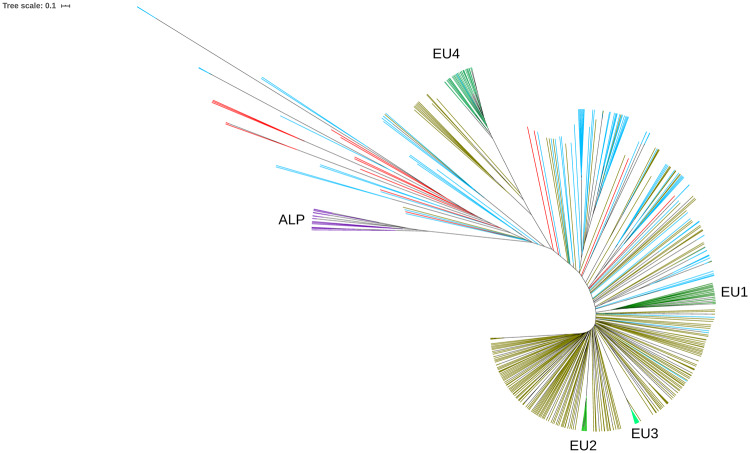
Phylogenetic tree of 500 *S. cerevisiae* strains produced from 16,672 SNPs using hierarchical clustering analysis showing the NZ *S. cerevisiae* wine group (blue), with previously identified wine group strains and EU subpopulations (green). The mosaic group (red) is characterized by admixture from other lineages. The neighboring alpechin group (purple), isolated from olive mill wastewater, is included to root the tree.

**Figure 2:**
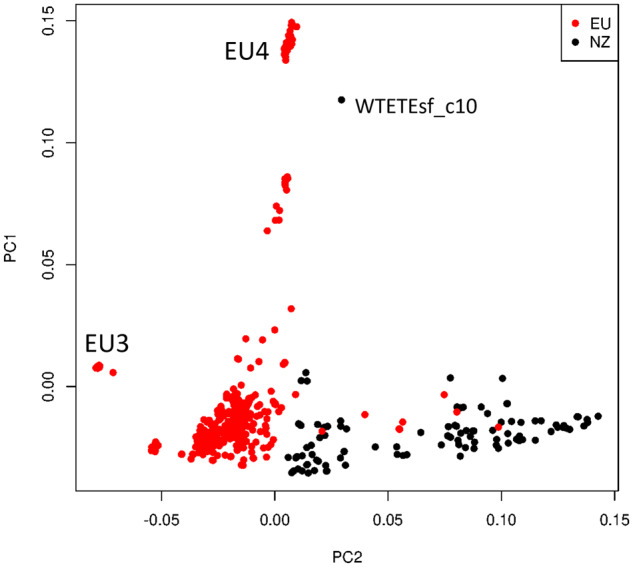
PCA of 16,672 SNPs from NZ derived and other wine group strains. PC1 and PC2 explain 2.86% and 2.34% of the variance. NZ samples are clearly distinguished from most wine group strains along PC2; however, eight American strains (7 Californian, 1 Argentinian) cluster with the NZ samples. PC1 primarily separates out two known EU subpopulations within the wine group, while EU1 and EU2 both form tight clusters within the majority of wine group strains. Two other groups are separate from main wine group cluster but do not correspond to previously identified subgroups. A single NZ outlier, WTETETsf_C10, is indicated.

**Figure 3:**
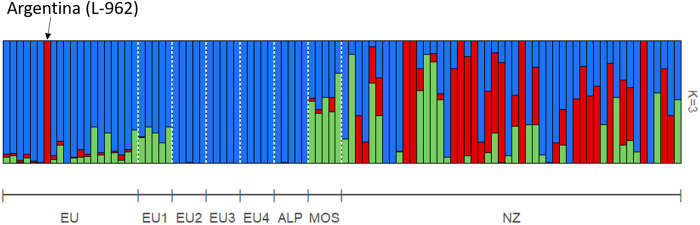
*Structure* analysis for *K* = 3 of 5 alpechin, 5 mosaic, 40 wine group (including 5 samples from each of the predefined EU1-4 subpopulations) and 50 NZ strains. The analysis converged with 200,000 iterations of burn in and 400,000 iterations of analysis. The EU subpopulations are highly homogenous, while mosaic and NZ populations appear admixed. In general, the wine group is far more homogenous than the NZ population; however, a single Argentinian sample is clearly distinguished.

An average of 44,960 homozygous and 6540 heterozygous SNPs was identified across the 104 NZ genomes when compared to the S288C reference, with a transition/transversion ratio of 2.6. When compared with the 1011 high-quality genomes from Peter *et al.* (2018), the position of NZ strains in the wider wine clade and global *S. cerevisiae* phylogeny is shown in Figure 1 and Supplementary Figure S1. Mosaic strains closely related to the wine group and characterized by admixture from other lineages (Peter *et al.* 2018) were also included in the wine-group analysis. NZ strains do not appear randomized among the wine group (green) but have a preponderance to cluster toward and with the mosaic clade at the root of the tree. To further elucidate the relationship of NZ strains, a PCA was performed on a subset of 16,672 SNPs, filtered to remove those in high LD (Figure 2). The first two principal components explained 2.86% and 2.34% of the variance and clearly separate the vast majority NZ and wine-group strains. PC1 primarily distinguished a number of apparent wine-group subpopulations and separated a single NZ sample, WTETETsf_C10, from the NZ group, possibly indicating the occurrence of more recent arrival [as has been shown previously by Goddard *et al.* (2010) via oak barrels from Europe] or admixture. PC2 distinguished the NZ group from all but eight of the wine-group samples. Further principal components were visualized but were less informative, with PC3 and PC4 explaining only 2.1% and 1.8% of the variance and failing to visibly distinguish wine and NZ strains. Structure analysis of a selection of 100 of these strains converged with 200,000 iterations of burn in and 400,000 iterations of analysis. The resulting inferred structure at *K* = 3 clearly distinguishes the majority of wine-group strains from the NZ population and correctly identifies the mosaic population as being highly admixed, and the EU subpopulations as more homogenous (Figure 3). The NZ samples do not form a single population but instead appear to be primarily the result of admixture between the European wine group and a second population. The Argentinian sample that clustered with NZ in the PCA is clearly distinguished as being a different population to the other wine-group samples.

### Phylogenetic analysis of Ty LTRs

Phylogenetic analysis using Maximum Likelihood (ML) was used to estimate the evolutionary history of each *Ty* family present in the NZ strains. Analysis of LTRs from SGRP strains of *S. cerevisiae* and *S. paradoxus* alongside those from the NZ population allowed the identification of ancestral shared insertions (*i.e.* those that occurred before NZ population isolation) and insertions unique to the NZ population occurring since colonization. No new evidence of horizontal transfer of *Ty* elements between species was recovered here. ML bootstrapping support for branches ranges from 0% to 100%mlBP (Maximum Likelihood Bootstrap) in each phylogeny; topologies are however plausible and consistent with previous results (Carr *et al.* 2012; Bleykasten-Grosshans *et al.* 2013). Returning poor support values is common for TE phylogenies, in particular LTR trees, regardless of inference method (*e.g.*Benachenhou *et al.* 2013).

**Figure 4:**
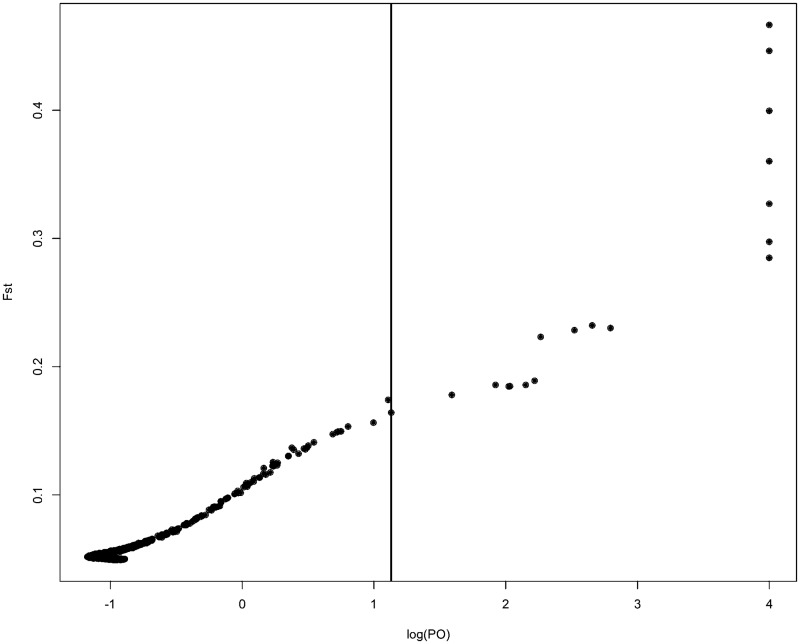
Maximum likelihood phylogeny using a nucleotide alignment of 353 *Ty1/2* LTR sequences analyzed at 337 sites. Strong or maximally supported (≥70% mlBP) branches are indicated by * where space allows (Ty1_2.tre file is available as file S1 in the Supplementary Material). Scale bar indicates number of substitutions per site/indicates that this branch has been arbitrarily shortened for figure clarity. *Saccharomyces* Genome Resequencing Project (SGRP; Liti *et al.* 2009) *S. cerevisiae* strains in red allow NZ LTRs (yellow) unique to the NZ population to be identified. The tree is rooted with *S. paradoxus Ty1* (brown) sequences. The presence of unique, recently active lineages (short-branched sequences) of *Ty1/2* hybrids and *Ty2* sequences in the NZ strains is indicated by black vertical bars. *Ty1/2* hybrid LTRs are likely the result of recombination between elements of the *Ty1/2* superfamily that share high nucleotide identity.

Figure 4 displays the *Ty1/2* superfamily phylogeny. Elements of the *Ty1/2* superfamily have been active in NZ populations since colonization (indicated by short-branched sequences), and *Ty1* activity has resulted in 18 unique solo LTRs (the result of LTR-LTR recombination) and three LTRs associated with full-length elements. Evidence of two highly active sub-lineages: *Ty1/2* hybrid and *Ty2* sub-lineage unique to the NZ strains (19 and 18 LTRs associated with internal coding regions, respectively) can also be observed (black vertical lines in Figure 4). The placement of *Ty2* within the *S. cerevisiae Ty1* clade is a known artifact: Carr *et al.* (2012) previously confirmed the origin of *Ty2* as a horizontal transfer event from *S. mikatae* into *S. cerevisiae*. *Ty2* sequences from *S. mikatae* were omitted here due to the focus on activity in NZ *S. cerevisiae*.

Supplementary Figures S2 and S3 display phylogenies for families *Ty3* and *Ty4*, respectively. The elements of both *Ty3* and *Ty4* have been minimally active since NZ colonization; *Ty3* possesses 25 short-branched unique insertions, although none of these are associated with full-length elements. As *Ty3* is active during meiosis (Bilanchone *et al.* 2015), these unique insertions may be indicative of the number of mating events having occurred in the NZ population since colonization. A further five long-branched *Ty3* insertions unique to NZ strains provide evidence of past activity of LTRs that have since accumulated mutations. *Ty4* is present in the NZ population with three unique short-branched insertions typical of relatively recent activity in the family, and four long-branched insertions displaying the accumulated mutations of aged insertions. The placement of the four long-branched NZ LTRs nested within *S. paradoxus* sequences may be the result of long-branched attraction, particularly given the poor support values for these branches (38%mlBP and 59%mlBP, respectively).

NZ strains possess only ancestral *Ty5* insertions (data not shown); therefore, it is likely that the extinction of this family in *S. cerevisiae* predates the isolation of the NZ population. Trees for *Ty1/2*, *Ty3*, *Ty4*, and *Ty5* are available in Supplementary Files S1–S4.

### Differentiating features of NZ isolates

**Figure 5:**
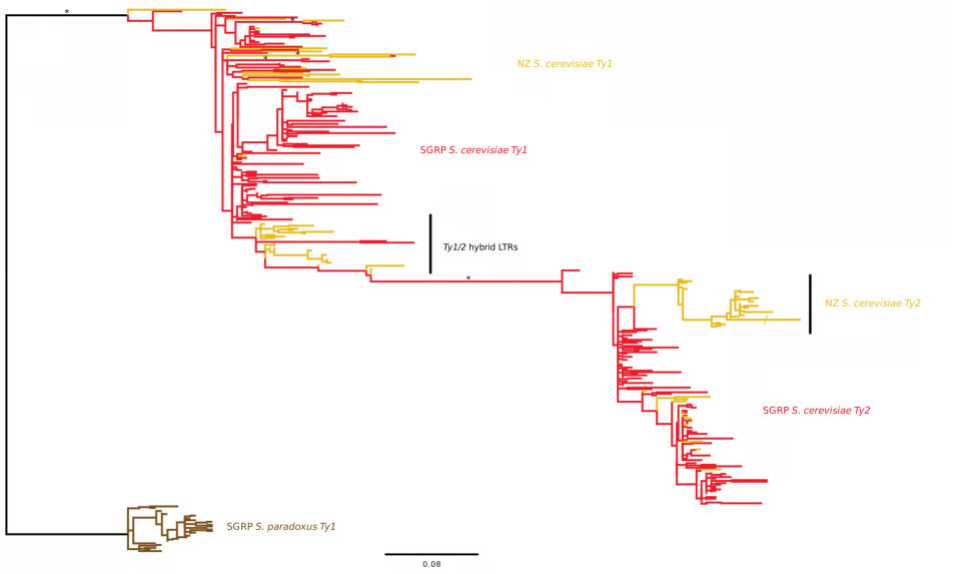
Posterior odds of being under selection (PO) is plotted against F_st_ for 7226 SNPs, measured between NZ and wine group *S. cerevisiae* strains. The vertical line represents a false discovery cutoff of 1%; SNPs to the right of this are identified as potential candidates for positive selection. Genomic information on these SNPs is provided in Table 2.

To identify candidate genes that may be under selection, Bayescan was used on a subset of 7226 SNPs from the combined wine-NZ data, filtered to remove rare SNPs, to detect SNPs with differing frequencies in the wine and NZ groups. Adequate convergence was achieved with the following parameters: “burn in: 100,000, thinning interval: 100, sample size: 5000, Nb of pilot runs: 200, length of each pilot run: 5000”. This was confirmed with Gelman and Rubin’s diagnostic, which gives a by-site point scale reduction factor average of 1.0 (max 1.06), indicating the variance between and within chains is approximately equal. Overall F_st_ between the populations were calculated at less than 0.05, indicating low differentiation (Hartl 1980). Using a strict false discovery rate of 1%, 18 candidate SNPs were identified (Figure 5). Seventeen of these SNPs were within coding regions of nine genes. Many of these SNPs are only short distances apart within each gene, and as such unlikely represent 18 unique fixation events but also some hitchhiking events. Six of the SNPs change amino acids within three proteins (OSW7, ELP2, and CWC22) associated with outer spore assembly, spliceosomes, and transcription (Table 1).

**Table 1:** Genomic location of 18 SNPs identified by Bayescan as candidates for selection based on differing allele frequencies between NZ and the wine group population

Chr	Location	Ref/SNP	Genome feature	Codon change	Synonymous	Function
1	138,667	A/G	XUT_1F-60	ncRNA	–	Xrn1-sensitive unstable transcript
2	372,349	C/T	TIP1	K/K	Y	Major cell wall mannoprotein
5	517,566	T/C	–	–	–	–
6	232,998	T/C	OSW7	N/H	N	Protein involved in outer spore wall assembly
6	233,004	T/C	OSW7	N/H	N	Protein involved in outer spore wall assembly
7	899,840	T/A	XUT_7F-413	ncRNA	–	Xrn1-sensitive unstable transcript
7	899,896	G/A	XUT_7F-413	ncRNA	–	Xrn1-sensitive unstable transcript
7	900,087	C/T	ELP2	E/E	Y	Subunit of Elongator complex
7	900,405	C/T	ELP2	L/L	Y	Subunit of Elongator complex
7	900,645	C/T	ELP2	L/L	Y	Subunit of Elongator complex
7	900,753	C/A	ELP2	E/E	Y	Subunit of Elongator complex
7	906,672	G/A	ELP2	S/S	Y	Subunit of Elongator complex
7	983,122	G/C	BRF1	S/F	N	TFIIIB B-related factor
7	1,048,402	G/A	CWC22	G/R	N	Spliceosome-associated protein
7	1,048,405	T/C	CWC22	S/P	N	Spliceosome-associated protein
7	1,048,409	G/A	CWC22	R/K	N	Spliceosome-associated protein
9	426,350	T/C	Ty1 LTR	–	–	Transposable element
10	114,907	T/C	JJJ2	Q/Q	Y	Protein of unknown function

The references allele is given for each SNP, along with the genomic feature containing the SNP and the identified function of this genomic feature. Coding SNPs are marked Y, and noncoding SNPs are marked N.

### Signals of selection within NZ

**Figure 6:**
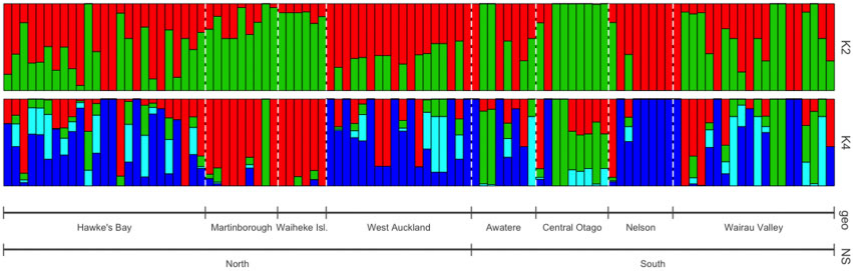
*Structure* analysis of 11,631 NZ SNPs using the admixture model with 200,000 iterations of burn in and 400,000 iterations of analysis for *K* = 2 and *K* = 4. The optimal number of populations was determined at *K* = 2 using the Evanno method; however, *K* = 4 was the next most optimal solution. Sampling location is strongly correlated with population assignment at both *K* = 2 and *K* = 4 (*P* ≤ 0.0001), but North/South Island origin is not (*P* = 0.4212).

Various SNP filtering levels were tested to reduce computational time and remove confounding and uninformative SNPs. The filters chosen reduced the total SNP count from 107,242 to 11,631, which were then used for analysis. The Linkage model in Structure failed to adequately converge with 10,000 iterations of burn in and 20,000 iterations of analysis and greater iteration numbers were not feasible due to the computational requirements. Under the Admixture model, convergence was achieved for *K* = 1 through *K* = 12 with 200,000 iterations of burn in and 400,000 iterations of analysis (Figure 6). Employing the Evanno method for calculating the most likely value of *K* indicated the optimal number of populations at *K* = 2; however, the graph also included smaller peaks at *K* = 4 and *K* = 6, indicating that these values are also plausible (Supplementary Figure S1). *K* = 3 and *K* = 5 produced multimodal results, indicating convergence to two different solutions, and as such were not analyzed further. ObStruct using Structure output at *K* = 2 and 8 sampling locations as predefined populations shows these to be strongly correlated (*R*^2^ = 0.39, *P* ≤ 0.0001) and using sampling locations with Structure output for *K* = 4 had a weaker correlation, though still highly significant (*R*^2^ = 0.27, *P* ≤ 0.0001). Using North/South islands as predefined populations found no correlation (*R*^2^ = 0.01, *P* = 0.4212).

**Figure 7:**
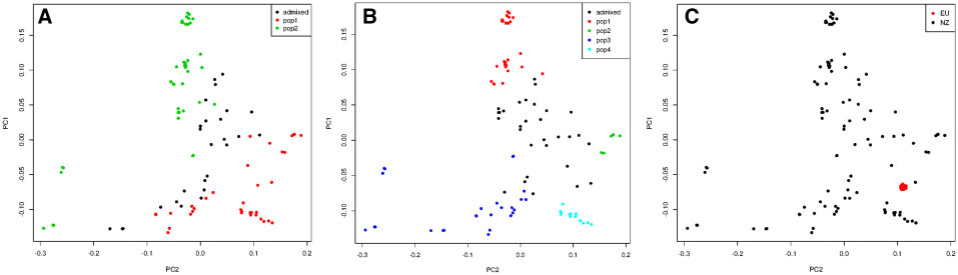
PCA of 8333 SNPs from NZ, showing how the first two principal components clearly distinguish assigned populations from structure for (A) *K* = 2 and (B) *K* = 4. Populations were assigned to strains with at least 0.8 inferred ancestry from that population. (C) Wine group strains form a tightly clustered group when projected on the NZ PCA.

PCA was used as an alternative method to evaluate the groups identified by Structure. After filtering for LD and missing rates, 8333 SNPs were kept for analysis from the NZ samples. Based on ancestry inferred with Structure for *K* = 4, 74 samples were assigned to four populations with 30 samples designated as admixed. PCA on these samples separated each of the four populations along PC1 and PC2, with admixed samples clustered in between (Figure 7b). PC1 and PC2 combined explain 9.99% of the variance and clearly separate the four populations and the admixed group. Additional PCs explained no more than 4% of the variance and were worse at distinguishing assigned populations. Assigning strains to the three populations based on the two modes found at *K* = 3 distinguishes similarly between populations 1 and 2, separated primarily along PC1, but varies in the assignment of populations 3 and 4. Using the inferred populations for *K* = 2 assigns 63 samples to populations, with 41 samples admixed (Figure 7a). These populations also separate along the two principal components but are less tightly clustered. Wine-group strains were projected onto the NZ principal components to compare the distinguishing features between NZ strains to the variation within the wine-group population. Here, they formed a tightly clustered group, distinct from any NZ subpopulations, indicating that the wine-group strains are largely homogenous for the features, which distinguish populations within NZ (Figure 7c).

### Copy number variants

A total of 4.6 million of reads mapped to the ORF pangenome. The median read depth per strain ranged from 20 to 75, with a median depth from all strains of 53. Of the 7796 ORFs in the pangenome, 6180 were present in NZ or wine-group strains to a median copy number of at least 0.5, with 5983 present in both populations. Of these, 1251 were enriched in NZ and 245 in the wine group, and 4487 had equal median copy numbers in both populations. Seventy-eight ORFs were enriched within NZ to an average copy number at least double that of the wine-group strains, and 1.26% of the total ORFs were present in both populations (Supplementary Table S2). GOrilla identified enriched terms for Process, Function, and Component (Table 2 and Supplementary Table S3). In total, 18 of these ORFs were associated with an enriched GO term. The most enriched terms were associated with four sugar transporter genes from the *HXT* family: *HXT1*, *HXT4*, *HXT7*, and *HXT16*. Attempts to identify ORFs with lower copy number in NZ relative to the wine group found numerous short, unverified ORFs with no GO terms associated, along with multiple mitochondrial ORFs. The 2μ plasmid was detected in every NZ strain, with an estimated median copy number of 47. In comparison, 331 of 362 wine-group strains contained the 2μ plasmid, with a median copy number of 11.5 when present.

**Table 2:** GO terms for genes enriched in NZ compared to the wine group when tested against a background of all identified *S. cerevisiae* genes in GOrilla

	*P*-value	Enrichment	Genes
Process			
Pentose transmembrane transport	1.80E−06	95.53	*HXT4*, *HXT7*, *HXT1*
Mannose transmembrane transport	5.65E−06	31.84	*HXT16*, *HXT4*, *HXT7*, *HXT1*
Carbohydrate import across plasma membrane	7.35E−06	29.97	*HXT16*, *HXT4*, *HXT7*, *HXT1*
Fructose transmembrane transport	9.39E−06	28.3	*HXT16*, *HXT4*, *HXT7*, *HXT1*
Import across plasma membrane	5.12E−05	18.87	*HXT16*, *HXT4*, *HXT7*, *HXT1*
Beta-alanine metabolic process	6.03E−05	127.37	*ALD3*, *ALD2*
Beta-alanine biosynthetic process	6.03E−05	127.37	*ALD3*, *ALD2*
Glucose import	1.02E−04	15.92	*HXT16*, *HXT4*, *HXT7*, *HXT1*
Hexose transmembrane transport	1.15E−04	15.44	*HXT16*, *HXT4*, *HXT7*, *HXT1*
Glucose transmembrane transport	1.15E−04	15.44	*HXT16*, *HXT4*, *HXT7*, *HXT1*
Monosaccharide transmembrane transport	1.15E−04	15.44	*HXT16*, *HXT4*, *HXT7*, *HXT1*
Carbohydrate transmembrane transport	1.30E−04	14.98	*HXT16*, *HXT4*, *HXT7*, *HXT1*
Polyamine catabolic process	1.80E−04	84.91	*ALD3*, *ALD2*
Carbohydrate transport	3.59E−04	11.58	*HXT16*, *HXT4*, *HXT7*, *HXT1*
Function			
Pentose transmembrane transporter activity	1.80E−06	95.53	*HXT4*, *HXT7*, *HXT1*
Mannose transmembrane transporter activity	4.26E−06	33.97	*HXT16*, *HXT4*, *HXT7*, *HXT1*
Fructose transmembrane transporter activity	4.26E−06	33.97	*HXT16*, *HXT4*, *HXT7*, *HXT1*
Glucose transmembrane transporter activity	5.65E−06	31.84	*HXT16*, *HXT4*, *HXT7*, *HXT1*
Monosaccharide transmembrane transporter activity	7.35E−06	29.97	*HXT16*, *HXT4*, *HXT7*, *HXT1*
Hexose transmembrane transporter activity	7.35E−06	29.97	*HXT16*, *HXT4*, *HXT7*, *HXT1*
Sugar transmembrane transporter activity	9.39E−06	28.3	*HXT16*, *HXT4*, *HXT7*, *HXT1*
Melatonin binding	3.68E−05	42.46	*ENO1*, *ENO2*, *ADH1*
Hormone binding	3.68E−05	42.46	*ENO1*, *ENO2*, *ADH1*
Carbohydrate: cation symporter activity	3.73E−05	20.38	*HXT16*, *HXT4*, *HXT7*, *HXT1*
Carbohydrate: proton symporter activity	3.73E−05	20.38	*HXT16*, *HXT4*, *HXT7*, *HXT1*
Carbohydrate transmembrane transporter activity	5.93E−05	18.2	*HXT16*, *HXT4*, *HXT7*, *HXT1*
Amide binding	1.11E−04	10.27	*ENO1*, *CPR1*, *ENO2*, *VTH2*, *ADH1*
Glyceraldehyde-3-phosphate dehydrogenase (NAD+) (non-phosphorylating) activity	1.80E−04	84.91	*ALD3*, *ALD2*
Solute: proton symporter activity	1.82E−04	13.77	*HXT16*, *HXT4*, *HXT7*, *HXT1*
Structural constituent of cell wall	1.82E−04	13.77	*TIR4*, *TIR1*, *TIP1*, *HSP150*
Solute: cation symporter activity	2.47E−04	12.74	*HXT16*, *HXT4*, *HXT7*, *HXT1*
Phosphopyruvate hydratase activity	3.58E−04	63.68	*ENO1*, *ENO2*
Symporter activity	4.27E−04	11.08	*HXT16*, *HXT4*, *HXT7*, *HXT1*
Component			
Cell periphery	2.37E−04	4.15	*TIR4*, *PHO5*, *HXT16*, *HXT4*, *TIR1*, *HXT7*, *TIP1*, *HXT1*, *YFL051C*
Fungal-type cell wall	3.42E−04	6.26	*TIR4*, *PHO5*, *PHO3*, *TIR1*, *TIP1*, *HSP150*
Phosphopyruvate hydratase complex	3.58E−04	63.68	*ENO1*, *ENO2*
External encapsulating structure	4.42E−04	5.97	*TIR4*, *PHO5*, *PHO3*, *TIR1*, *TIP1*, *HSP150*
Cell wall	4.42E−04	5.97	*TIR4*, *PHO5*, *PHO3*, *TIR1*, *TIP1*, *HSP150*

GO terms for Process, Function, and Component were tested separately, and the *P*-values and enrichment values are presented here. For additional details of GO enrichment, see Supplementary Table S3.

## Discussion

Previous research on NZ *S. cerevisiae* has disagreed on the position of the NZ *S. cerevisiae* subgroup within the wine yeast clade (Goddard *et al.* 2010; Cromie *et al.* 2013; Gayevskiy *et al.* 2016). This study provides some evidence for the placement of the NZ *S. cerevisiae* population as a recently diverged population from the rest of the wine yeast clade (see Figure 2): they are clearly genetically similar to the established wine group but tend to be placed more basally. However, whether the NZ strains form a single clade is less clear, as phylogenetic approaches give less clarity (Figure 1). This is not surprising given the admixed nature of the NZ population (Figure 3), and the flawed assumptions of the hierarchical clustering used, which ignores this. Regardless, this study provides further support for a historic range expansion of *S. cerevisiae* that correlates with European colonists to NZ, followed by subsequent diversification and admixture. This provides clear evidence for the range expansion of this species, but as with any other study that infers past migration from current taxa, it cannot be determined with a high degree of certainty if the genomic features distinguishing NZ and wine-group populations were present in the strain(s), which originally colonized NZ, or arose through adaptive or neutral evolutionary processes thereafter.

The population structure of *S. cerevisiae* within NZ is best explained with two subpopulations; however, PCA implies that four subpopulations may be present. This suggests that after a colonization event, dispersal into a new habitats/ranges may have has been constrained to some extent and resulted in subpopulation. Given the difficulties achieving convergence and the effect that filtering had on preliminary analysis, it is hard to determine the precise number of subpopulations. Previously, 16 populations were inferred using microsatellite data at 8 loci from 369 *S. cerevisiae* isolates using Instruct (Gao *et al.* 2007; Knight and Goddard 2015). As this study includes only 104 strains, it is possible that some of the original populations were absent or underrepresented. However, given the far greater number of loci used in this analysis, 8333 as opposed to 8, the resulting population structure will be better resolved. It has been demonstrated that SNP data is significantly better at detecting admixed populations and it is possible that some admixed populations were incorrectly identified as unique subpopulations (Gärke *et al.* 2012). The populations found here are structured based on sampling location, agreeing with previous studies both in NZ and in European vineyards (Capece *et al.* 2016). There was no evidence of the Cook Strait presenting a genetic barrier between North and South Island, as has been found in several invertebrate species (Trewick *et al.* 2011). Both the presence of the 2μ plasmid in all NZ strains and itshigher average copy number compared to other wine-group strains imply more frequent sexual cycles have occurred to maintain it (Futcher *et al.* 1988), and this is in line with the inference of an outcrossing rate between 11%-42% from 850 NZ strains (Knight and Goddard 2015). There appeared to be lower copy numbers of mitochondrial genes in NZ; however, this is highly related to growth conditions and not informative of genetic differences between strains (Rafelski *et al.* 2012).

The evolutionary history of retrotransposons typically reflects that of their host on an individual basis (Bowen *et al.* 2003) and as a host population (Hosid *et al.* 2012). While previous research has explored the activity and insertion patterns of *Ty* families in multiple populations of *S. cerevisiae* (Carr *et al.* 2012; Bleykasten-Grosshans *et al.* 2013), the work here presents the first known phylogenetic analysis of LTRs within the isolated populations of NZ. Transposition activity of families *Ty3-5* reflects that of previous research; however, the divergence and high level of activity of *Ty2* in an isolated population is yet to be reported. *Ty2* is known to be a relatively recent addition to the *S. cerevisiae* genome, likely gained by horizontal transfer from *S. mikatae* (Liti *et al.* 2005; Carr *et al.* 2012; Naseeb *et al.* 2017), which clearly predates the colonization of NZ and may account for *Ty2*’s relative high activity levels. Czaja *et al.* (2020) have recently shown that elements of the *Ty1-2* superfamily have a complex history and further research is required to understand how *Ty* elements contribute to the diversity of the *S*. *cerevisiae* genome.

Comparing ORF copy number between wine-group and NZ populations reveals a significant amount of variation. The gene ontology of the most enriched ORFs indicates that specific sugar transporter genes have increased in copy number in NZ. Expansion of *HXT7* has previously been demonstrated in experimental evolution studies under glucose limitation (Brown *et al.* 1998; Kao and Sherlock 2008), along with contraction occurring under high glucose concentrations (Wenger *et al.* 2011). Other *HXT* genes enriched are involved in transportation of different sugars and may be expanded or contracted under high or low sugar concentrations. As such, this result correlates with the observation of routes to adaptation in short-term experimental studies and suggests an adaption toward altered sugar conditions in NZ compared to other wine-group strains. *S. cerevisiae* has been isolated from NZ soils and native forests (Knight and Goddard 2015) and one explanation is that this represents an adaptation to habitats other than grape juice. These *HXT* genes represent only 4 of 78 ORFs expanded in NZ, and only 18 of those 78 were associated with enriched GO terms. Further investigation of these other enriched ORFs may reveal additional environmental adaptations in *S. cerevisiae*; however, we suggest that those 60 ORFs not associated with enriched GO terms may result from neutral processes.

Overall, it is clear that the *S. cerevisiae* population in NZ has a number of genetic features differentiating it from the closely related EU population and is reasonably the result of microbial range expansion. While it is impossible to determine if these features have arisen since colonization or were present in the *S. cerevisiae* strain(s), which founded the NZ population, some appear to have possible adaptive value. Within NZ, *S. cerevisiae* is highly geographically structured at a local level, and this structure is determined by patterns of genetic variation not found within wine-group strains. It is evident that the colonization of NZ has produced a distinct group of *S. cerevisiae* wine-group strains, providing further support for theories of microbial range expansion emphasizing non-environmental factors. The difference in frequencies of a number of synonymous SNPs indicates that much of the genomic variation has arisen by neutral processes, and while evidence for adaptive selection may be seen in copy number variation, it is unclear if the majority of genes enriched in NZ arose from adaptive processes. Although this study has determined some of the genomic changes this colonization has caused within *S. cerevisiae* in NZ, it has also identified a number of NZ and American strains, which are differentiated from other wine-group strains by various genomic features. These may warrant further investigation to determine how these differences arose, the relationship of these American strains to the NZ population, and if the novel genetic diversity of these may contribute to ongoing work aimed at improving wine production.
