# Corrigendum to: Whole-genome sequencing from the New Zealand *Saccharomyces cerevisiae* population reveals the genomic impacts of novel microbial range expansion

**DOI:** 10.1093/g3journal/jkab135

**Published:** 2021-06-18

**Authors:** Peter Higgins, Cooper A Grace, Soon A Lee, Matthew R Goddard

*G3: Genes|Genomes|Genetics*, 11, 2021, https://doi.org/10.1093/g3journal/jkaa027

In the article by P. Higgins, C. A. Grace, S. A. Lee, and M. R. Goddard (*G3: Genes|Genomes|Genetics* 11(1)) entitled “Whole-genome sequencing from the New Zealand *Saccharomyces cerevisiae* population reveals the genomic impacts of novel microbial range expansion”, on pages 6-7 the images for Figure 4 and Figure 5 were reversed. These images now appear above the correct caption. In addition, in the final paragraph on page 4 the sentence “Supplementary Figures S2 and S3 display phylogenies for families Ty3 and Ty4, respectively.” has been corrected to read “Supplementary Figures S3 and S4 display phylogenies for families Ty3 and Ty4, respectively.”

All results and conclusions of the manuscript remain unchanged.

